# Robust Data Association Using Fusion of Data-Driven and Engineered Features for Real-Time Pedestrian Tracking in Thermal Images

**DOI:** 10.3390/s21238005

**Published:** 2021-11-30

**Authors:** Mircea Paul Muresan, Sergiu Nedevschi, Radu Danescu

**Affiliations:** Computer Science Department, Technical University of Cluj-Napoca, 28 Memorandumului Street, 400114 Cluj Napoca, Romania; Sergiu.Nedevschi@cs.utcluj.ro (S.N.); Radu.Danescu@cs.utcluj.ro (R.D.)

**Keywords:** data association and tracking, convolutional neural networks, feature engineering, thermal imaging, autonomous driving, advanced driving assistance systems

## Abstract

Object tracking is an essential problem in computer vision that has been extensively researched for decades. Tracking objects in thermal images is particularly difficult because of the lack of color information, low image resolution, or high similarity between objects of the same class. One of the main challenges in multi-object tracking, also referred to as the data association problem, is finding the correct correspondences between measurements and tracks and adapting the object appearance changes over time. We addressed this challenge of data association for thermal images by proposing three contributions. The first contribution consisted of the creation of a data-driven appearance score using five Siamese Networks, which operate on the image detection and on parts of it. Secondly, we engineered an original edge-based descriptor that improves the data association process. Lastly, we proposed a dataset consisting of pedestrian instances that were recorded in different scenarios and are used for training the Siamese Networks. The data-driven part of the data association score offers robustness, while feature engineering offers adaptability to unknown scenarios and their combination leads to a more powerful tracking solution. Our approach had a running time of 25 ms and achieved an average precision of 86.2% on publicly available benchmarks, containing real-world scenarios, as shown in the evaluation section.

## 1. Introduction

Multiple Object Tracking (MOT) is one of the most fundamental problems that have been addressed in computer vision and robotics. Tracking is an important building block in various tasks of computer vision such as surveillance [1], autonomous driving and advanced driver assistance systems [2], or industrial inspection [3]. Even though it has attracted the interest of many researchers over several decades, the problem of multiple object tracking has not yet been solved. Many of the MOT methods follow a track by detection framework where the tracking solution generally employs an object detector to identify objects in each frame and then utilizes an association method between detections and tracks, in order to maintain their identity over all frames from a given image sequence. MOT can be separated into Online and Offline tracking methods according to how they use object detection information in the image sequence. Offline methods [1,4] handle the tracking problem as a global optimization problem and make use of all detections available from the whole image sequence when associating unique track identities to these detections. Therefore, offline methods can only be applied when the whole image sequence is present. In contrast, online methods are more suitable for real-time applications since they rely on the information from object detection up to the current frame. These real-time solutions have also shown competitive tracking accuracy on international benchmarks [5,6].

The challenges that appear in multi-object tracking can be split in two main categories: sensor-related issues and data association problems. Some of the thermal sensor issues may refer to:

- The number of objects within the field of view (FOV) of the sensor, which may be unknown and in different states.
- Objects enter and leave the sensor FOV; therefore, it is necessary to have good object management and object identity management.
- Since the object detector is not perfect, it may be susceptible to two kinds of errors, missed detections (due to environment conditions, object properties, or occlusions) and false detections or clutter (a detection that is not caused by an object). Both types of errors could lead to disastrous outcomes if they are not handled correctly.

The main idea of the data association problem is that there is no information regarding the origin of a detection or what real object caused it. Hence, we can split the challenges for treating the data association problem into two categories:

- The origin uncertainty: There is no knowledge about how the new measurements relate to previous sensor data, and
- Motion uncertainty: Objects can have multiple motion patterns, which may change in consecutive frames.

The poor handling of the data association problem may lead to bad tracking results. The issues mentioned above were approached by many researchers who have addressed the tracking problem for different kinds of applications using different types of sensors such as single cameras [7], stereo cameras [8], LIDARs, RADARs [9], or thermal cameras [10]. Some solutions from the literature try to improve the performance of object tracking by fusing the information from multiple sensors [9]. 

Even so, to ensure high-quality results and robustness against individual sensor failure, the tracking functionality must be reliable and the solution must not be centered around the functioning of a certain sensor.

Thermal cameras have attracted a lot of attention in the automotive field due to their ability to detect objects in bad weather conditions including rainy, snowy, or foggy weather. Other advantages of thermal cameras include their ability to function without a light source, the lack of saturation in the presence of the lights from oncoming vehicles, and the ability to detect people or animals from long ranges even at night, improving the reaction time of the driver. The main disadvantages of thermal images are that they do not contain as much information as the color or even the monochrome images, and they usually have a lower resolution, which makes the design of a data association function based on appearance even more difficult. There are two main directions in the literature for addressing this issue of data association: the feature engineering approaches [10,11,12,13,14,15,16,17,18,19,20,21] and the data-driven methods (using convolutional neural networks) [22,23,24,25,26,27,28,29,30,31]. The advantage of designing the data association function using data-driven methods is that after the convolutional neural network architecture is designed, through a learning process the best features are identified. The main issue with deep learning and with data-based models in general is that the object tracker may get latched onto the wrong object, which may be a false detection but looks similar to data from the training dataset, and never recover. Furthermore, if the data association model is not trained on parts of objects, the tracker can have a hard time tracking an object when it is partially occluded. 

In contrast to data-driven methods, in the feature engineering-based solutions the researchers manually design features and cost functions and use an optimization method [13] to assign the best measurement to each track. The difficulty in this approach is identifying the best features to use for each type of sensor. Feature engineering methods are faster than data-driven solutions; however, identifying the correct features to use depending on the sensor is a more difficult endeavor.

In this paper we present a data association and tracking solution for thermal images that exploits the benefits of both approaches. The proposed tracker was designed to track pedestrians in thermal images related to traffic scenarios. The contributions of this paper are the following.

- We designed a family of five Siamese Convolutional Neural Networks that were combined to create a data-driven, appearance-based association score capable of working even in the case of partial occlusions. The base architecture of all neural nets is similar and its design is also a contribution of the current paper.
- We proposed a uniform, local binary pattern descriptor obtained from edge orientations. This engineered feature will be used to compute a similarity score between measurements and tracks. The number will be included in the data association score to provide adaptability to unknown scenarios.
- The creation of the dataset is useful for training a CNN when designing an appearance data association function for tracking pedestrians in thermal images. The dataset is made publicly available.

The data-driven and feature engineered scores were merged using a weighted combination and the resulting number was used to perform a successful data association and track objects.

The rest of the paper is structured as follows. In Section 2 we present the state of the art. In Section 3 we describe the proposed contributions. In Section 4 we illustrate the performance of the proposed solution, and in Section 5 we conclude the paper.

## 2. Related Work

In this section we will review state-of-the-art methods that address the problem of tracking using convolutional neural networks and feature engineering methods. 

### 2.1. Feature Engineering-Based Tracking Methods

Most online tracking algorithms use a tracking-by-detection approach, where a detector provides the object candidates and, using a data association function, measurements and tracks are correlated across multiple frames. When computing the similarity cost between detections in different frames, object appearance and motion are the most common sources of information. In appearance-based cost computing, some traditional methods use the distance computation between color histograms [7]. A similar approach was presented in [11], where the similarity measure was calculated using the Chi-Square similarity of the gray level histograms of the object and track and the cosine distance of the spatio-temporal location of the two compared entities. The authors in [12] engineered an appearance similarity cost function using multiple types of information including object dimension and color histogram. Additionally, they used the L2 norm to compute the motion similarity between detections and tracks, and then fused the results with the score obtained from the appearance function. The authors calculated the association scores for all measurements and all tracks and then used the Hungarian algorithm [13] to find the best mappings. In the work presented in [14], the authors engineered an aggregated local flow descriptor that encodes the relative motion pattern of two bounding box detections in different time frames. The descriptor was used along with other features to find the best data association between targets and detections. The authors of [10] designed a cost function where they used a combination of multiple features such as HOG, width, height, and intersection over union between the measurement and track bounding boxes in order to create an efficient data association and tracking approach for objects detected in thermal images.

Bertozzi [15] applied a stabilization technique to cope with vehicle movements affecting camera calibration. Localization and tracking of the pedestrians were based on the search for warm symmetrical objects that had a specific aspect ratio and size. Other approaches track pedestrians using hot areas. For example, the HotSpot tracker detects objects by performing a pixel intensity thresholding and tracks the detections using a Kalman filter with a global nearest neighbor approach to the association problem [16,17]. The paper in [18] presented a weighted function that combines similarities in position, size, and appearance. The main issue with this work is that the appearance score was computed in a naive manner and, in the case of pedestrian overlapping in some situations, the data association may fail. Yu et al. [19] used edges and edge orientations and transferred them into the Fourier domain to obtain a real-time tracker. Another tracker that was applied on thermal images [20] used edge features and a 2640-dimensional histogram feature computed from the intensity channel. In [21] the authors combined a motion and an appearance score for improving the data association process from the tracking framework. The appearance cost, between the track and measurement, was composed of a weighted combination of multiple individual scores obtained via feature engineering. Some of the quantities used were the mean, standard deviation in the region of interest, the height, width, classification score, the uniform LBP of grayscale values from the regions of interest, and an intersection over union score. The motion cost, between a track and a detection, included the Euclidean distance in position between the two objects, a deviation cost, which illustrated the drift in the motion pattern of the current measurement, and an optical flow cost. The combination of the motion and appearance costs led to the creation of an efficient tracker.

### 2.2. Data-Driven Tracking Methods

In the recent literature, common approaches for trackers were to model object features using deep convolutional neural networks (CNNs). In the approach presented in [22], to ensure robustness against background noise in the case of online training of CNNs, the TCNN algorithm maintained stability of appearance through a tree structure of CNNs. SRDCFir [23] is the adaptation of the SRDCF tracker for thermal images. This tracker introduces a spatial regularization component that penalizes filter coefficients residing outside the target region, leading to a more discriminative appearance model. In addition to the HOG features used in [24], the SRDCFir employs channel-coded intensity features and a motion feature channel. 

Recently, an idea that became popular in visual object tracking, which also obtained competitive results on international thermal imaging benchmarks, used a pre-trained function to verify the level of similarity between measurements and tracks [25]. The matching function is usually implemented by a two-branch CNN, whose branches are the same and share the parameter space between them. The Siamese network takes the image pairs (from the track and measurement) as input and outputs the similarity between them. In the work of Liu et al. [26], the authors trained a multi-layer fusion Siamese network to learn the similarity of two arbitrary objects from thermal images using flow information. The presented network had multiple convolution layers and attempted to fuse deep layers and shallow layers to obtain richer information for the data association function. Zhang et al. [27] proposed a multi-stage deep feature fusion network, which combined a multi-stage region proposal network (RPN) based on one-stage RPN and a spatial transformer network for tracking objects in thermal images. SiamFC [25] is another tracker that uses Siamese Networks, which can run in real time; however, its tracking accuracy is inferior to state-of-the-art trackers, due its lack of online adaptation ability. The DSiamM [28] tracker proposes to make an online update to the Siamese network by integrating correlation filters into the network. 

The solution presented in [29] decomposes the robustness and discrimination requirements in separate stages. In their approach, the authors addressed each stage by training one network. Furthermore, for strengthening the robustness of their solution, two Siamese AlexNet [30] networks were used for feature extraction and, finally, the results obtained from each stage were fused in order to create an efficient data association function. In [31], Zhang et al. proposed a method of generating a thermal imaging data set from a RGB data set. Using this data set, the authors performed an end-to-end training using a Siamese neural net model [32] for obtaining the thermal image features. The obtained features were used for computing the similarity between objects in the data association function. 

We built upon the state of the art by creating a data association solution that efficiently combines the data-driven and feature-engineered costs in order to create a robust data association function useful within the tracking framework. We used the motion and appearance scores presented in [21] and we added to the appearance score two additional terms. The first term was a feature engineered score that was derived by combining the uniform LBP with HOG features, and the second term was a data-driven term obtained by using a family of Siamese neural networks. The architecture of a Siamese neural network is also an original contribution of this paper. The model was trained using the dataset presented in Section 3.2.4, which has been made publicly available. The combination of the feature engineered and data-driven costs led to a solution, which is more robust and is capable of tracking objects even in scenarios where the usage of a individual type of cost failed. The mentioned contributions are detailed in Section 3. In Table 1 the main differences of the proposed solution, with respect to some methods from the state of the art, are presented. The “x” mark from a table cell refers to a specific feature of the method. 

## 3. Proposed Solution

### 3.1. Camera Setup

To ensure that our solution was able to accurately track pedestrians in various scenarios, we recorded sequences in all weather and illumination conditions (day, night, rain, sun, snow, fog, etc.). The thermal imaging sensor used consisted of a FLIR PathFindIR, which incorporated a Vox microbolometer with a spectral response in the ranges of 8–14 µm. The sensor can output images having a resolution of 320 × 240 pixels and it was equipped with a 19-mm lens providing a field of view of 36° (h) and 27° (v). The camera can perform in various weather conditions while being protected from dust or water due to the fact that it is hermetically sealed (IP67 rated). The thermal time constant of the used thermal camera is 12 ms.

**Figure 1:**
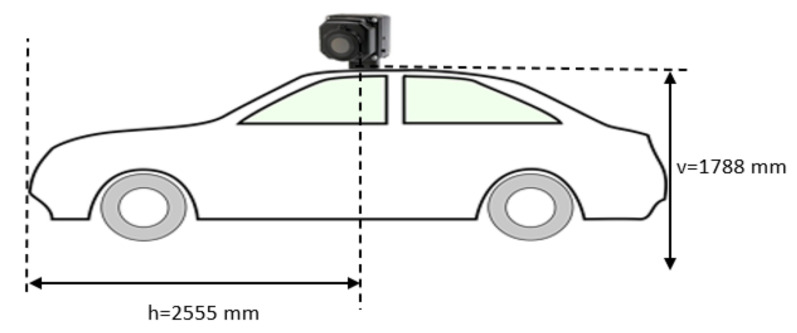
Camera position and mounting on the vehicle.

The camera outputs its data in analog format (PAL), which is converted to digital using the DVD EZMaker 7 converter from AVerMedia. The converted images were upscaled to a 640 × 480 resolution. The camera was fixed on top of the vehicle, at an equal distance to the lateral sides of the vehicle, using a magnetic mounting tripod. The mounting and position of the camera on the vehicle can be seen in Figure 1. On the horizontal axis the position of the camera on the car was 2555 mm, and on the vertical axis the camera was mounted at a height of 1788 mm.

### 3.2. Proposed Approach

In this paper, we build upon the solution presented in [21], which was considered the base solution for our approach. In this section we are providing some details regarding the base solution and in the following subsections we describe the proposed contributions. It is worth mentioning the fact that the techniques presented in this paper can be applied to other tracking frameworks as well to improve the overall data association and tracking performance.

The proposed solution followed a tracking-by-detection framework, where the similarity cost function between a track and a detection includes both motion and appearance scores. The input of our algorithm was given by a set of bounding boxes, and the output was a set of tracks that had a smoothed trajectory and unique ID. The high-level modules from the processing pipeline of an autonomous vehicle or advanced driving assistance systems can transform the results of the tracking algorithm into an actionable output or warning message for the driver.

The main components of the tracking solution included the following modules: clutter elimination, similarity cost computation, track and detection association, track update, and results’ refinement. For reducing the running time of the association process between a track and a detection, a validation gate was used around the position of the predicted hypothesis. The detections that fell within the validation gate of a track were considered in the association process of that specific track. The tracks and detections were associated using a similarity cost function based on appearance and motion.

The appearance score is useful in target tracking for differentiating between objects using visual features. Furthermore, the appearance score should adapt to the changes that appear in consecutive frames for the same instance due to deformations or point-of-view changes. In thermal images, distinguishing between objects can be particularly difficult, in comparison to RGB images, because of the lack of color information or relevant texture information. The appearance score, between a track *i* and a detection *j*, onto which we built our current solution contained several visual features, as illustrated in Equation (1).

(1) $$\vartheta \left(i,j\right)=w_{hL}hL\left(i,j\right)+w_{\mu s}\mu s\left(i,j\right)+w_{\sigma s}\sigma s\left(i,j\right)+w_{hs}hs\left(i,j\right)+w_{ws}ws\left(i,j\right)+w_{cs}cs\left(i,j\right)+w_{os}os\left(i,j\right)$$

In Equation (1) above, $hL\left(i,j\right)$ represents the difference between the histogram of uniform local binary pattern (LBP) in the region of interest (ROI) of the detection j and track i, $\mu s\left(i,j\right)$ is the mean value pixel intensity distance of the ROI, $\sigma s\left(i,j\right)$ represents the variance score in the ROI, $hs\left(i,j\right)$ and $ws\left(i,j\right)$ are the differences in height and width between the track *i* and detection *j*, $\sigma s\left(i,j\right)$ represents the overlapping distance, and $cs\left(i,j\right)$ represents the class detection probability score. Additionally, to the appearance score, a motion score was been used. The expression of the motion score between the track i and detection j is given by Equation (2).

(2) $$m\left(i,j\right)=w_{dst}dst\left(i,j\right)+fc\left(i,j\right)+w_{\sigma m}\left(\sigma m{\left(i,j\right)}_{x}+\sigma m{\left(i,j\right)}_{y}\right)$$

The meaning of the terms used are: $dst\left(i,j\right)$ is the euclidean distance between the track and detection position; $fc\left(i,j\right)$ is the difference in the optical flow in the regions of interest, between the track i and detection *j*; and $\sigma m{\left(i,j\right)}_{x}\wedge \sigma m{\left(i,j\right)}_{y}$ are the scores that illustrate the deviation of the object’s motion from the motion pattern it had so far, on the x and y axes.

The weights introduced in both Equations (1) and (2) allow us to set the influence of certain parameters. Their value was determined experimentally and can be found in [21]. The final similarity cost was composed of the sum of the motion and appearance costs.

The similarity costs between tracks and all the detections that fell within their covariance ellipses were stored in memory and were fed to an optimal assignment algorithm [13] to find the best correspondences. The following three scenarios can be identified after running the Hungarian algorithm: We can have a track matched with a detection, an unmatched detection, or an unmatched track. Each of these scenarios are addressed separately and they are presented in Section 3.2.3.

In this section we describe the proposed contributions and how they were used to improve the data association and tracking performance. First, in Section 3.2.1, we will present the proposed family of Siamese Neural Networks used for obtaining the data-driven score. Secondly, we present the novel feature engineered descriptor in Section 3.2.2. In Section 3.2.3, we detail how the proposed data associations’ scores were included in the tracking framework, and, finally, in Section 3.2.4, we will detail how we created the pedestrian dataset and what this dataset contained.

#### 3.2.1. Data-Driven Score

Creating a data association function that can be used to track objects can be addressed using similarity learning. We proposed to learn a function, $\gamma \left(i,j\right)$, that compared a given thermal image, which belonged to the measurement *j*, to a candidate image, which had the same size and belonged to track *i*, and returned a high score if the two images were different and a small score otherwise. In this section we will discuss implementing the function $\gamma \left(i,j\right)$ using a deep convolutional network.

Similarity learning, in the context of CNNs, is typically addressed using Siamese architectures, which apply the same transformation φ to both input images and then combine their results using a function *g,* as shown in Equation (3). If we consider the function *g* a distance or similarity metric, the function φ can be considered an embedding.

(3) $$\alpha \left(i,j\right)=g\left(\varphi \left(x\right),\varphi \left(y\right)\right)$$

**Figure 2:**
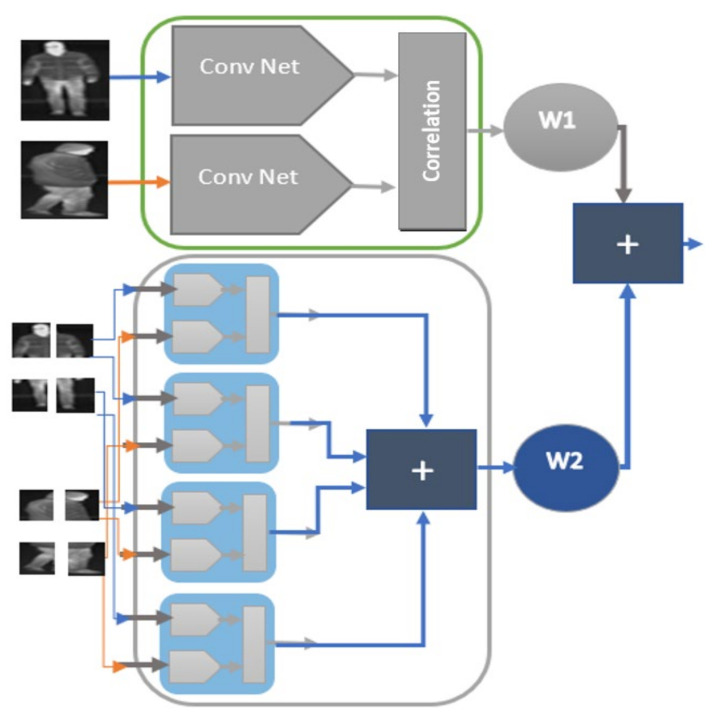
Architecture of the proposed data driven cost based on a family of Siamese Networks. These networks work on the whole image and on parts of it, improving the robustness of the TIR tracker.

To obtain a more effective appearance score for thermal image tracking, we constructed a family of Siamese networks. We called the proposed Siamese networks a family of networks due to their similar structure. Unlike existing solutions based on Siamese networks, which often compute the similarity using the entire detection, we computed the similarity using multiple networks trained on the whole detection and also on parts of it, as depicted in Figure 2, which made our solution more robust in cases of occlusion.

To this end, we designed two different types of network structures: the first type of network model will work on the entire detection, while the second model will work on parts of the detection. The proposed network models will work on detections having a dimension of a minimum (width × height) of 19 × 50 pixels. For dimensions smaller than the ones mentioned, the data association will work using only the feature-engineered score. The first step in our solution was to resize the input representing the detected image rectangle to a size of 200 × 200 pixels. Thermal infrared emission does not depend on any light source; however, the emissivity of the clothes that each person wears leads to a unique thermal texture and structure for each pedestrian. Even though the environment in which the target is plays a large role in the apparent temperature of the target (the at-aperture-measured target radiance is a function of the emissivity of the target, the reflectivity of that same target, and the thermal environment that the target is in), the tracking algorithm is not drastically affected by this aspect because the frame rate of the camera is sufficiently large and the characteristics of each track are updated at each frame using the features of the detections. The characteristics of the same target do not change drastically between frames; so, the data association function can make the right correspondences.

**Figure 3:**
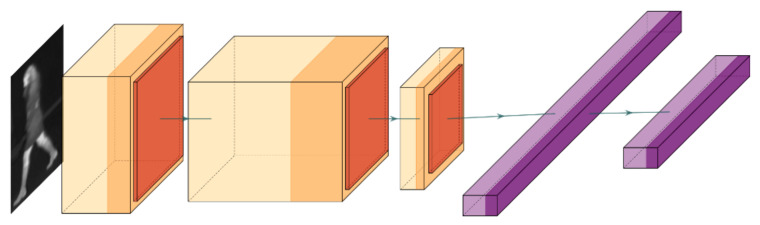
Graphical depiction of the embedding function φ used for generating the features for the input image.

In the second step, to compute the texture and structure appearance similarity, we designed a CNN able to capture the changes in appearance and the texture uniqueness of each pedestrian such that the tracker was able to distinguish easily between objects. The architecture that we adopted for the embedding function φ consisted of eight layers, as shown in Figure 3. Specifically, we first used a convolutional layer with a kernel size of 3 × 3 and 96 filters. Then, we used a ReLU activation followed by a max pooling layer and a dropout of 25%.

The second convolution layer used the kernel size of 3 × 3 and 128 filters, and similarly to the previous case, this layer was followed by a ReLU activation and a max pooling layer with a 25% dropout. The final convolutional layer used a kernel size of 3 × 3 having 12 filters and ReLU activation and it was followed by the max pooling and dropout with a 25% dropout rate. The last two layers were fully connected layers: the first layer having a size of 128 nodes and a ReLU activation followed by a 10% dropout, and the second layer having 50 nodes and ReLU activation. The embeddings of the two images were compared using the Euclidean distance. To train the neural net, we used the contrastive loss function (4), where Y is the tensor of details about image similarity, which is 0 if the inputs are from the same class and 1 otherwise, *D* is the tensor of Euclidean distances between the pairs of images, and margin is a constant used to enforce a minimum distance between them. In our scenario, it had a value of 1.

(4) $$Loss=\frac{YD^{2}+\left(1-Y\right)\max{\left(margin-D,\;0\right)}^{2}}{2}$$

For creating the training dataset for the part-based model, the original image was split into four equal parts, i.e., top left, the top right, the bottom left, and the bottom right part. The part-based similarity networks were trained on parts of the image. There was one Siamese Network responsible for identifying the similarity between each of the four parts from the target with the corresponding part of the measurement. The function $h\left(x_{p},y_{p}\right)$ (Equation (5)) that computed the similarity between parts *p* of the *x* and *y* images was defined similarly as the function presented in Equation (3); however, the embedding function φ was different for the part-based scenario. The overall similarity was computed by summing the scores obtained for each part, as shown in Equation (6).

(5) $$h\left(x_{p},y_{p}\right)=g\left(\varphi_{p}\left(x_{p}\right),\varphi_{p}\left(y_{p}\right)\right)$$

(6) $$\beta \left(i,j\right)=\overset{4}{\underset{p=1}{\sum }}h\left(x_{p},y_{p}\right)$$

The part-based models were also trained using contrastive loss and had similar architectures to the model created for the entire image; however, the number of nodes of the last fully connected layer, number of filters, and kernel sizes were different. The part-based models had 30 nodes for the last fully connected layer and the kernel size of all layers was 3 × 3, while the number of filters for the first convolutional layer was 112, second layer convolutional layer was 96, and the number of filters for the third convolutional layer was 12. The final data association score of the data-driven component was computed, as described in Equation (7), where *w*_1_ is 100 and *w*_2_ is 25, are two weights that were determined experimentally.

(7) $$\gamma \left(i,j\right)=\alpha \left(i,j\right)w_{1}+\beta \left(i,j\right)w_{2}$$

#### 3.2.2. Feature Engineered Score

Object texture did not change drastically between frames; therefore, it is a good feature to use to measure the correlation between track and measurement. We aimed to better capture the texture structure of each object by creating a feature that combined the histogram of oriented gradients’ descriptor and the uniform local binary pattern descriptor. Furthermore, using an engineered feature we made the proposed tracking method more adaptable to unknown scenarios. For computing this descriptor, we first computed the magnitude G and the orientation θ of the gradient using the input images derivatives $I_{X}$ and $I_{Y}$ (8). The image was split into cells having a dimension of 10 × 10 pixels. For each cell a nine-bin histogram was created and every pixel from that cell cast a weighted vote in the histogram based on the orientation of the gradient of that pixel, with the weight being the magnitude of the gradient.

(8) $$\left|G\right|=\sqrt{I_{X}^{2}+I_{Y}^{2}};\theta =arctan\left(\frac{I_{X}}{I_{Y}}\right)$$

We then iterated each cell from the image, and assigned for that cell the orientation corresponding to the bin that has the largest value from the histogram. To the obtained result, the local binary pattern (LBP) descriptor was applied, Equation (9).

(9) $$LBP_{P,R}=\overset{P-1}{\underset{p=0}{\sum }}s\left(g_{p}-g_{c}\right)2^{p}$$

The number of neighbors for a pixel in a neighborhood of radius R is the value P, and the function s is defined as *s*(*x*) = 0 if x > 0 or *s*(*x*) = 1, otherwise. In our scenario, $g_{p}$ was the orientation of neighbour pixel *p*, and $g_{c}$ was the orientation of the center pixel. In the proposed solution, a neighborhood of 3 × 3 was used; hence, all values from the region of interest could be represented using a 256-value histogram. In order to improve the memory consumption and the running time and to achieve more robustness against noise, a uniform local binary pattern histogram was employed [34]. Therefore, for the proposed neighborhood, there were 256 possible patterns, out of which 58 were meaningful; hence, there were a total of 59 bins necessary.

**Figure 4:**
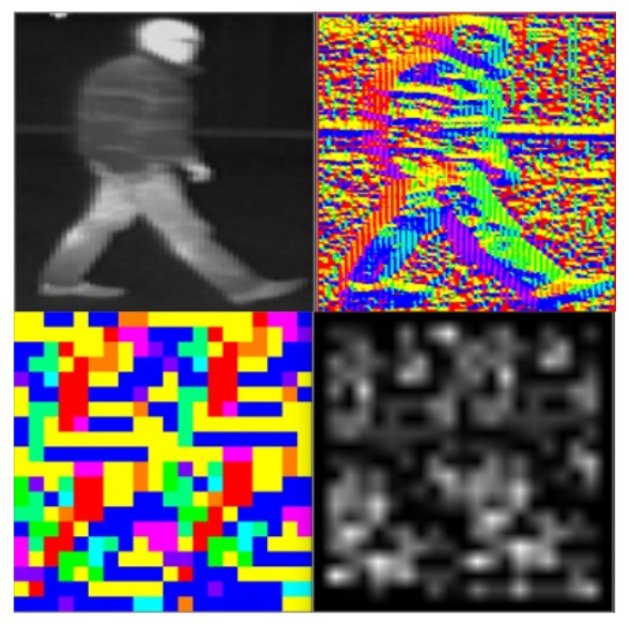
Top left image is the original image. The top right image represents the orientation of each pixel, which is represented using a different color. In the bottom left, the dominant orientation for each cell of 10 × 10 pixels is selected. In the bottom right image, the LBP representation of the orientation image is shown. Using the information from this LBP image, a uniform local binary pattern histogram is created.

The voting of each LBP code was done using a lookup table to improve the running time of the solution. The resulting histogram was denoted as θLBP. A graphical depiction of the main steps can be seen in Figure 4. After resizing the original image, some artefacts may appear in the image and look like vertical stripes. These artefacts do not affect the overall performance of the algorithm.

The final similarity score between the input image belonging to the track *i* and measurement *j*, with respect to the proposed feature, was computed using the root mean square function on the values of the histograms, θLBP, for the track *i* and measurement *j* (Equation (10)).

(10) $$\tau \left(i,j\right)=\sqrt{\frac{1}{59}\overset{59}{\underset{k=1}{\sum }}{\left(\theta LBP{\left(k\right)}_{i}-\theta LBP{\left(k\right)}_{j}\right)}^{2}}$$

#### 3.2.3. Data Association Score and Tracking

The appearance score between track *i* and measurement *j*, using both the engineered feature and the data-generated features, is given by Equation (11). The value of $w_{3}$ is 300 and was determined experimentally by performing extensive tests on multiple scenarios. The term $\vartheta \left(i,j\right)$ was introduced in Equaiton (1). It is worth mentioning that all the weights used in our solution were stable with respect to the test data. They did not require modifications when the scenarios were changing or when using other thermal images acquired with the same sensor.

(11) $$\mu \left(i,j\right)=\vartheta \left(i,j\right)+w_{3}\tau \left(i,j\right)+\gamma \left(i,j\right)$$

An optimal assignment algorithm [13], is used to find the best correspondences between the tracks and measurements from the current frame. After the optimal assignment, the following scenarios were encountered: a track matched with a measurement, an unmatched track, and an unmatched measurement. In the case of a successful track measurement assignment, the track and all its parameters are updated with the new information coming from the measurement. In the case of an unmatched measurement, a new track is created, which will remain in an unstable state until it will be tracked for another five frames and, afterwards, will become stable and will be displayed.

One of the key features of tracking is the persistence of a tracked object even if it goes undetected or occluded for a number of frames. For this reason, the proposed tracker maintained a history counter that counted the number of frames for which a track is not associated. The position of the track in future frames was predicted using the motion pattern the tracked object had so far. After a number of frames, if the track remains un-associated, it entered a drifting stage where it was not displayed anymore, but it was kept in memory. The track was finally removed when, in the drifting stage, it was not associated with any new measurements.

Therefore, the tracks that were stable and were not associated for a number of frames were not removed immediately. The tracked objects were updated and new positions were predicted using the Kalman Filter [2].

**Figure 5:**
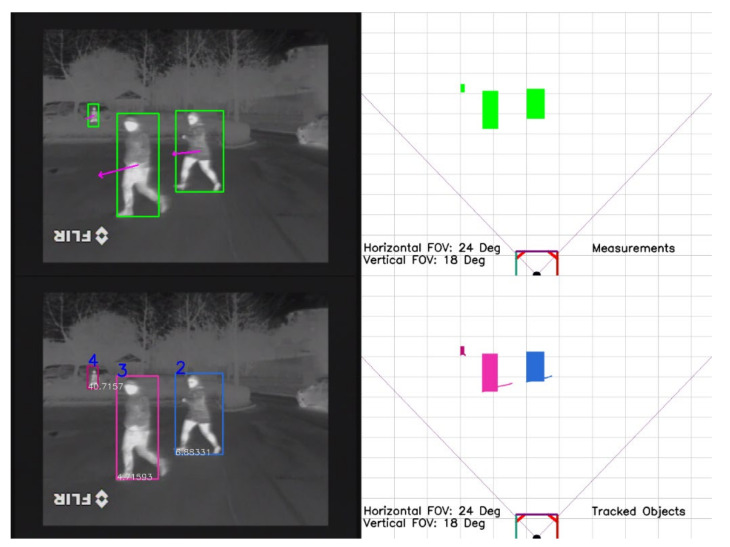
Pedestrians in a parking lot. The proposed tracking solution is able to track pedestrians of different sizes, even when they are partly occluded.

The track history counter threshold used in the proposed solution was 20 and the drifting history counter threshold was 15. If a track was created and not updated for five frames, it was removed immediately. In Figure 5, we show a scenario where two pedestrians are tracked as they are heading towards their vehicle, and a third pedestrian, in the background, is tracked even when he/she is partly occluded by vegetation. The bottom right image from Figure 5 shows the past position of each pedestrian path. The bottom left image shows the tracked objects with their corresponding unique ID. The top left image illustrates the measurements as they are detected by the object detector, and in the top right image the corresponding measurements are projected in a virtual image.

**Figure 6:**
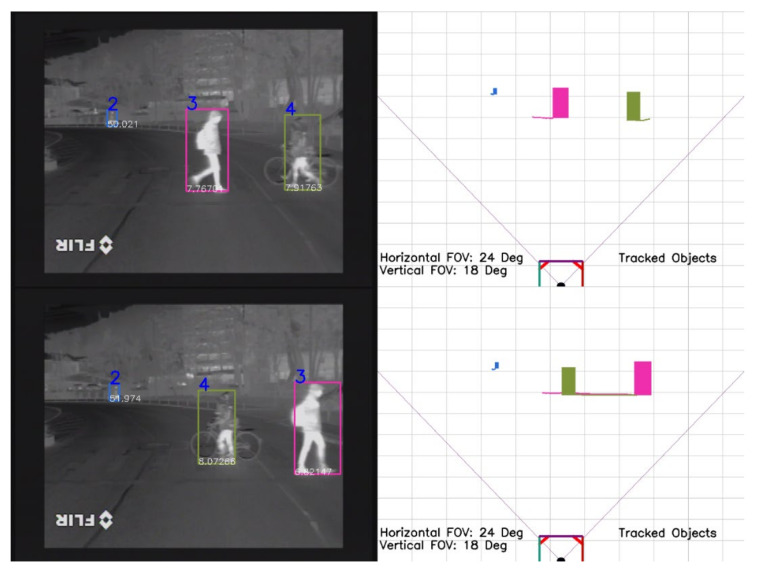
Pedestrians overlapping as they are crossing the street. The tracker is able to maintain the correct object ID and not latch onto the wrong object. Both the top two and bottom two images represent the same tracked objects seen at different time stamps.

The bounding box of each object has a unique color to highlight its unique identity. In Figure 6, we illustrate another scenario in which two people cross paths. Even when the two pedestrians overlap, the proposed tracking solution is able to maintain the correct identity of each pedestrian and not latch onto the wrong pedestrian.

**Figure 7:**
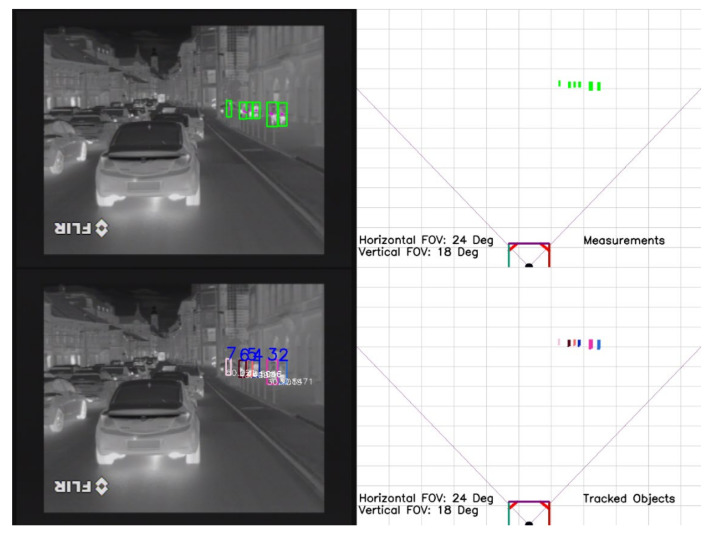
Multiple pedestrians are tracked. No ID switch appears among the tracked objects.

In Figure 7, multiple pedestrians are tracked as they are walking on the sidewalk. Even though the pedestrians are close to each other and they are getting smaller as they are going further from the ego vehicle, no ID switch appears among the tracked objects. The meaning of the four images presented in Figure 7 remains the same as in Figure 5.

#### 3.2.4. Pedestrian Dataset from Thermal Images

**Figure 8:**
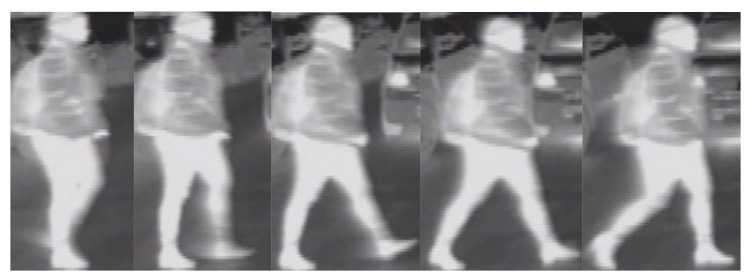
Sample images from the created dataset extracted for the same pedestrian instance.

Siamese networks extract features from data pairs and generate embedding vectors, which can be compared using an energy function in order to verify the similarity between the input pair. In order to train our Siamese networks to differentiate between pedestrians, we created a dataset consisting of over 200 pedestrian instances that were cropped from thermal image sequences. The dataset contains over 26,000 images of pedestrians captured in different weather and light conditions. The conditions in which the sequences were recorded were specific to driving scenarios and included scenarios for day, night, rain, fog, clear weather, spring, and winter. The pedestrians were extracted from the recorded thermal image sequences in three passes. In the first pass, each pedestrian was cropped from all frames from each sequence, and the cropped images were stored in a folder having the sequence name. Then, in the second pass, all pedestrian images that represent the same object instance were grouped in folders. Since some consecutive sequences may have contained the same instances of pedestrians, in the third and final pass, we cross-checked all folders from all sequences and placed similar pedestrian instances from different sequence folders in the same pedestrian instance folder from one of the sequences. We finally placed all the pedestrian instance folders in a data set folder and we gave each one an order number. Some samples from the pedestrian data set from thermal images corresponding to the same pedestrian instance are displayed in Figure 8. This dataset can be used to train data-driven models in order to aid the pedestrian reidentification (data association) process in tracking applications. Table 2 shows the attributes of the created dataset. The reason why some of the images may seem to have a lower resolution compared to the images from other thermal cameras [35] is that the cropped pedestrians can be farther away from the vehicle-mounted thermal camera. The created dataset can be downloaded from the link https://users.utcluj.ro/~mmp/DatasetPaper/ (latest accessed on 29 November 2021)

## 4. Results

The proposed tracking framework was implemented using C++ and Python, and all test cases presented in this section were done on a computer having an Intel i7-4770 K CPU with 3.5-GHz frequency and 8 GB of RAM memory and the GPU used was NVIDIA GeForce GTX 1080 Ti. The designed tracker was able to track pedestrians having an average running time on the CPU and GPU of 25 ms (without the object detection part). The proposed data-driven score was implemented on the GPU, while the feature engineered score was implemented on the CPU.

For training the neural networks, the proposed dataset, presented in Section 3.2.4, was used. Furthermore, the original dataset was augmented using the following operations: image flip, adding salt and pepper noise in the image, addition of motion blur, addition of gaussian noise, image sharpening, and contrast normalization. The resulting dataset was split for training the proposed neural network architectures in the following way: 20% test data, 10% cross-validation data, and 70% training data. Each model was trained for 40 epochs using a learning rate of 0.0005 and the optimizer used was root mean square propagation. The results of the proposed models on the test sets were the following: 98.34% for the model working on the entire image, 96.82% for the neural network working on the top left image part, 96.61% for the neural network model working on the top right part of the image, 95.92% for the bottom left part, and 96.01% for the bottom right part. The object detector employed in our solution was a YOLO [36]-based detector, which was trained on the FLIR-ADAS [37] dataset and fine-tuned on the CrossIR [21] dataset obtained with a PathFindIR thermal camera. The CrossIR dataset contains images taken in various light conditions (day and night) and different weather conditions (sunny, rainy, foggy) and temperature conditions (cold and warm).

**Figure 9:**
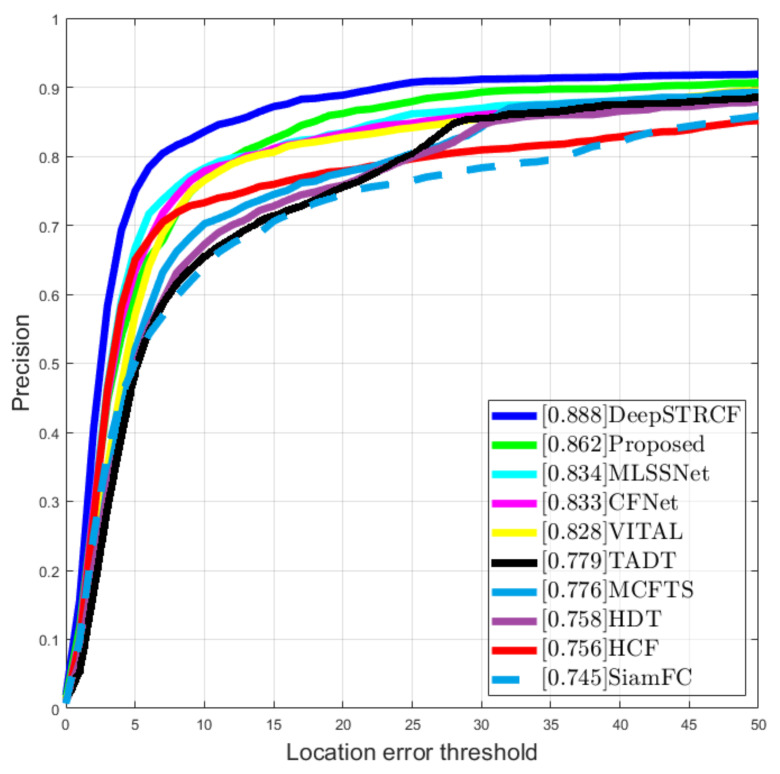
Position precision plot on the PTB-TIR benchmark.

**Figure 10:**
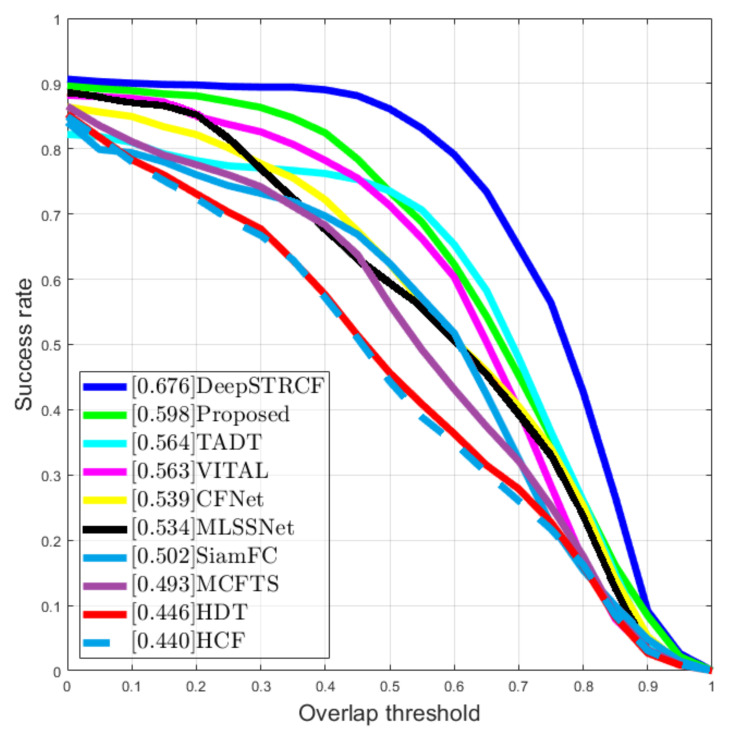
Plot that measures the overlapping score between the tracked object and ground. truth.

We compared the performance of the proposed tracker with other state-of-the-art solutions using the PTB-TIR benchmark [38]. In this dataset, there are multiple image sequences acquired using a thermal camera, each having manual annotations. One comparison metric used in this dataset was the center location error (CLE), which is defined as an average Euclidean distance between the object position and ground truth position for that object. If the CLE is within a given threshold (20 pixels on the PTB-TIR benchmark), the track is said to be successful at that frame. Furthermore, the benchmark also offers results from multiple types of trackers on the given sequences such that the advantages and disadvantages of each method can be studied comparatively. In the evaluation of the proposed tracker on the PTB-TIR benchmark, we included only the sequences that were acquired from a vehicle-mounted camera, since the target application of our solution was related to intelligent vehicles. The evaluation result of the proposed solution with respect to the CLE metric on the all the automotive sequences from the benchmark is displayed in the precision plot in Figure 9. The numerical results and plots from both Figure 9 and Figure 10 were obtained using the PTB-TIR Evaluation Toolkit, which is presented in detail in [38].

For better visibility. the values illustrated in Figure 9 are also displayed in Table 3.

Another interesting score that the PTB-TIR benchmark provided was the overlap score, which measures the overlap ratio between the bounding box area of the tracked object and the ground truth. The tracking is labelled successful at that frame if the overlap score is above a threshold. The success plot is used to rank the tracks with respect to their overlapping score at the threshold varying from 0 to 1. In Figure 10, the success plot is displayed.

In contrast to the top solutions from this benchmark, our method was designed keeping in mind the constraints of the automotive field. The proposed solution was able to track objects even in occluded scenarios, and in the case of an unknown environment situation, which was not present in the training set, the method was able to track the object detections. Moreover, the proposed approach was able to perform multiple-object tracking not just single-object tracking.

Furthermore, the proposed solution is not very complicated to reproduce, does not require huge amounts of data for training, and can be easily augmented with other features.

We also display the values from Figure 10 in Table 4 for better visibility.

Additionally to the evaluation metrics presented above, we also evaluated the proposed solution using the MOTA (multi-object tracking accuracy) and MOTP (multi-object tracking precision) metrics. The equation for the MOTA is presented in Equation (12) and for MOTP in Equation (13).

(12) $$MOTA=1-\frac{\sum_{t}\left(FP_{t}+FN_{t}+IDSW_{t}\right)}{\sum_{t}GT_{t}}$$

(13) $$MOTP=\frac{\sum_{t,i}d_{t,i}}{\sum_{i}c_{t}}$$

The MOTA metric serves as a general error rate for trackers that takes into account all object configuration errors that were made by the tracker, like false positives, misses, mismatches, and over all frames. The maximum MOTA achievable is 1, which would indicate that a tracker has no errors. The second metric, MOTP, evaluates the precision of the bounding boxes. Between all track hypotheses and ground truth bounding boxes a distance metric is computed and divided by the number of matched objects to compute an average precision. These values are then summed over all frames from the testing sequence to compute the MOTP. The essential difference between the two metrics is that MOTP takes into account bounding box accuracy over time for tracked and matched objects, while MOTA summarizes tracking errors over time, including tracks that go unmatched. An IDSW(id switch) occurs when a track is lost and re-initialized with a new id or when the object identity is incorrectly swapped because of a wrong track and detection association. In Table 5 we illustrate the evaluation using the MOTA, MOTP, and IDSW of the proposed tracker in the context of multiple pedestrian tracking on the CrossIR dataset [21].

The proposed solution was able to accurately associate detections to tracks and perform multiple pedestrians’ tracking in thermal images regardless of the weather conditions or if the object became occluded. By combining the data-driven and feature engineered scores, we ensured that the tracker could adapt to unknown traffic situations, thus becoming more robust.

To illustrate how much the proposed tracker improves the detection process, we will define several metrics. We say that an object is correctly identified if its position differs from the position of the ground truth with at most 10 pixels (on the x or y axis). Precision is defined as the number of correctly identified objects divided by the number of total objects from the ground truth for a frame. Recall is the number of correctly identified objects divided by the number of total detected objects for that frame. The accuracy of the tracker and detector is defined as the number of correctly identified objects reported to the number of total objects from the ground truth. The detector and the tracker were evaluated on over 100 sequences having multiple objects, which contained different weather and lighting conditions obtained from real traffic scenarios.

The evaluation presented in Table 5 was performed on the CrossIR dataset introduced in [21]. We performed this evaluation to illustrate the performance of the proposed algorithm in the presence of multiple objects, in various weather conditions. It is a known fact that object detectors may fail to detect some objects when they are occluded or because of the accuracy of the detector. In this evaluation we aimed to illustrate the fact that the object tracking is improving the overall detection of pedestrians, being able to maintain an identified object even when the object detector is not able to accurately identify a pedestrian.

The comparative evaluations are presented in Table 6. The proposed method was built upon the base solution presented in [21]. In Table 6, we made an ablation study and show the performance of the base solution and each of the proposed contributions individually. We also illustrate the fact that the results obtained using the fusion of the proposed data-driven and the feature engineered costs, added to the base solution, improve the tracking performance in all the metrics presented below.

As can be seen, the proposed solution improved the performance of the object detector, leading to better overall results. Furthermore, it is worth noting that the feature engineered score can also be applied to other object classes, such as vehicles; but, illustrating this was out of the scope of the paper.

## 5. Conclusions

In this paper, we presented a novel data association solution useful in multi-object tracking, which can efficiently track pedestrians in thermal images. To address the main issues of the data association problem in thermal images, we created a hybrid data association function that fuses data-driven scores with feature engineered scores in order to obtain a high-quality and adaptable tracking approach. Specifically, we created a family of five Siamese Neural Networks that were trained on the image boxes corresponding to the detected objects and on their parts, which generated similarity scores for input images. The data-driven similarity scores between the detected objects and the tracks were obtained using a weighted combination between the scores from the Siamese Networks. Furthermore, to better capture the texture of objects and make our solution more adaptable to unknown scenarios, we introduced a descriptor that encapsulates the edge information in a uniform, local, binary pattern histogram that can be used to compare the objects’ interest. The final appearance score from the data association function combined the feature engineering and the data-driven score to create a robust tracker for objects in thermal images. We also created and made publicly available a pedestrian dataset from thermal images, which can be used for training data-driven models to learn features from these images. The proposed approach obtained 86.2% precision on the PTB TIR benchmark and ran in 25 ms, achieving real-time performance. In future approaches, we will work on improving the quality of the proposed tracker by automatically finding the weighting parameters, used when combining features, in an unsupervised manner for each feature.

## Figures and Tables

**Table 1:** Differences of the proposed solution with respect to some methods from the state of the art.

Method	Feature Engineered	Data Driven	Whole Detection	Part-Based
Online Tracker [18]	x		x	
Tracker [21]	x		x	
SiamFC [25]		x	x	
MLSSNet [33]		x	x	
**Proposed Solution**	x	x	x	x

**Table 2:** Attributes of the constructed dataset.

Attribute	Value
Video Sequences Used	160
Total Extracted Image Samples	26,153
Pedestrian Instances	207
Camera Position	Vehicle Mounted
Pixel Resolution	8 bpp
Original Image Resolution	640 × 480

**Table 3:** Evaluation with respect to the precision metric.

Method	Tracking Precision Score
DeepSTRCF [23]	88.8%
**Proposed**	**86.2%**
MLSSNet [33]	83.4%
CFNet [32]	83.3%
VITAL [39]	82.8%
TADT [40]	77.9%
MCFTS [41]	77.6%
HDT [42]	75.8%
HCF [43]	75.6%
SiamFC [25]	74.5%

**Table 4:** Evaluation with respect to the success score.

Method	Tracking Success Score
DeepSTRCF [23]	67.6%
**Proposed**	**59.8%**
TADT [40]	56.4%
VITAL [39]	56.3%
CFNet [32]	53.9%
MLSSNet [33]	53.4%
SiamFC [25]	50.2%
MCFTS [41]	49.3%
HDT [42]	44.6%
HCF [43]	44%

**Table 5:** Evaluation with respect to different metrics.

Method	MOTA	MOTP	IDSW
**Proposed**	**86.14%**	**88.63%**	**134**
Base Solution	81.36%	83.17%	143
TADT	80.3%	81.7%	121
MLSSNet	79.8%	82.3%	269
SiamFC	76.4%	82.1%	343

**Table 6:** Ablation study with respect to several metrics.

	Average Precision	Average Accuracy	Average Recall
Object Detector	75.98%	66.47%	98.67%
Base Solution	80.01%	76.4%	95.8%
Base with only Data-Driven Score	86.15%	79.22%	93.21%
Base with only Engineered Score	83.25%	78.43%	93.71%
**Base with all Fused Scores (proposed)**	88.61%	80.02%	94.8%

## Data Availability

The dataset that resulted from this study can be found at the link: https://users.utcluj.ro/~mmp/DatasetPaper/. For evaluating the solution proposed in this paper we have used the PTB-TIR dataset and Evaluation Toolkit which can be found at the link: https://sites.google.com/view/ptb-tir.
